# Energy Saving Effects of Wireless Sensor Networks: A Case Study of Convenience Stores in Taiwan

**DOI:** 10.3390/s110202013

**Published:** 2011-02-10

**Authors:** Chih-Sheng Chen, Da-Sheng Lee

**Affiliations:** 1 Graduate Institute of Mechanical and Electrical Engineering, National Taipei University of Technology, No.1, Sec. 3, Zhongxiao E. Rd., Taipei City, 106, Taiwan; 2 Department of Energy and Refrigerating Air-Conditioning Engineering, National Taipei University of Technology, No.1, Sec. 3, Zhongxiao E. Rd., Taipei City, 106, Taiwan; E-Mail: f11167@ntut.edu.tw

**Keywords:** WSN, PMV, CFD, marginal energy conservation benefit

## Abstract

Wireless sensor network (WSN) technology has been successfully applied to energy saving applications in many places, and plays a significant role in achieving power conservation. However, previous studies do not discuss WSN costs and cost-recovery. The application of WSNs is currently limited to research and laboratory experiments, and not mass industrial production, largely because business owners are unfamiliar with the possible favorable return and cost-recovery on WSN investments. Therefore, this paper focuses on the cost-recovery of WSNs and how to reduce air conditioning energy consumption in convenience stores. The WSN used in this study provides feedback to the gateway and adopts the predicted mean vote (PMV) and computational fluid dynamics (CFD) methods to allow customers to shop in a comfortable yet energy-saving environment. Four convenience stores in Taipei have used the proposed WSN since 2008. In 2008, the experiment was initially designed to optimize air-conditioning for energy saving, but additions to the set-up continued beyond 2008, adding the thermal comfort and crowds peak, off-peak features in 2009 to achieve human-friendly energy savings. Comparison with 2007 data, under the same comfort conditions, shows that the power savings increased by 40% (2008) and 53% (2009), respectively. The cost of the WSN equipment was 500 US dollars. Experimental results, including three years of analysis and calculations, show that the marginal energy conservation benefit of the four convenience stores achieved energy savings of up to 53%, recovering all costs in approximately 5 months. The convenience store group participating in this study was satisfied with the efficiency of energy conservation because of the short cost-recovery period.

## 1. Introduction

Wireless sensor networks have been developed for many applications [1–3], including home energy conservation, environmental monitoring, and machinery sensor monitoring. Sensors produced by semiconductor manufacturing and integrated-circuit manufacture have the advantage of low-cost [4], making possible the application of wireless sensor networks to energy conservation problems. Wireless sensor networks that implement intelligent surveillance and energy management can contribute to achieving an intelligent and comfortable shopping space and help attain the goal of intelligent living technology.

Three technological innovations, namely integrated circuit (IC) sensors, wireless communication technology, and energy harvesting chips are a key to the success of these applications. IC technology has greatly reduced the cost of manufacturing sensors. Wireless communication technology can obviate the inconvenience created by the cable configurations of sensors, allowing sensor networks to be more practicable [5–9]. The stray energy in convenience stores includes thermal, vibration, and indoor light sources. As Table 1 shows, the highest energy density involves light sources [10–12], therefore, these are the main focus of this study. Energy harvesting chips [14] convert light sources into effective electric power that drives distributed sensors. This design also helps prevent pollution from disposed batteries. A photovoltaic cell with a harvesting chip, IC sensor, and wireless communication components can create a “deploy and forget” sensor network that efficiently decreases maintenance and operation costs.

The WSN is an indispensable element in achieving the energy conservation goals. In this study, several sensors scattered throughout the space of a convenience store were connected to a network to detect physical environmental measurements, air conditioning control parameters, and the status of power distribution panels and customers as a basis for energy conservation.

Previous studies have shown that in light powered wireless networks the cross correlation between the results of Predicted Mean Vote (PMV), an index of human thermal comfort, and a comfort sensing vote by people is as high as 95% [13]. After installing the WSN, air conditioning a convenience store using the rules that PMV provides can achieve optimal operation for energy saving control. This method also achieves the highest energy efficiency, at 54% [14,15]. The successful energy conservation strategy in this study is based on an understanding of the usage and status of equipment power consumption.

## 2. Wireless Sensor Network

**Figure 1.**
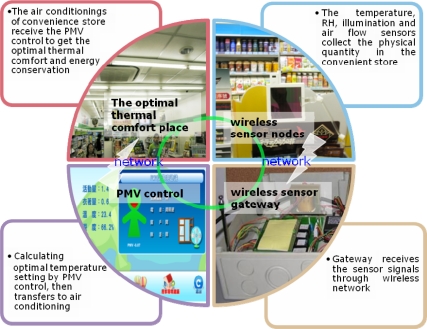
The schematic view of WSN to air conditioning in convenience stores: wireless sensor nodes, wireless sensor gateway, PMV control.

The thermal flow sensor network in convenience stores combines the thermal comfort of man (Man) and air conditioning (Machine) to achieve adaptively-adjusted energy management that can establish instant communication and control [5,16–18] and achieve an optimal thermal comfort level. The common sensor types for convenience stores include temperature, relative humidity, air flow, and illumination sensor nodes. The framework of a sensor star network includes sensors and a gateway located on the store ceiling. Using wireless communication, sensor nodes pass information to a gateway and a computer then calculates the range of PMV index, identifying temperature and fan speed modulation for achieving optimal air conditioning control. Figure 1 shows the logical control of the air conditioning energy conservation system.

### 2.1. Sensor Nodes Wireless Charging and Sensor Components

**Figure 2.**
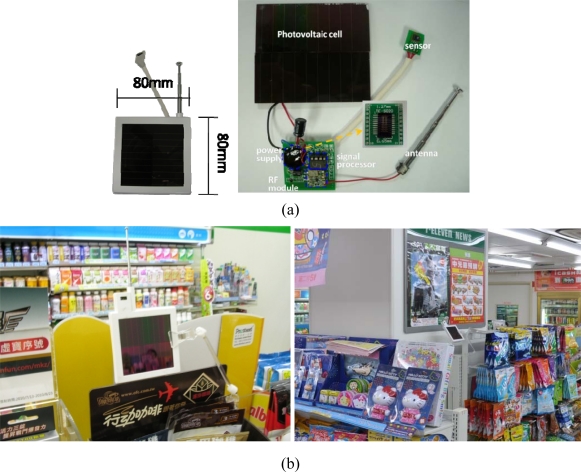
(**a**) Structure of wireless sensor node [14]; (**b**) Sensor node distribution in a convenience store.

A convenience store is a typically bright location that is able to provide enough light power to charge powerless wireless sensor nodes, allowing them to collect and transfer information, as Figure 2 shows. Photovoltaic cells provided power to the sensor nodes. Under exterior light irradiation, each photovoltaic cell produced an open-circuit voltage of *V_oc_*; the voltage source connected the load and work producing close-circuit current was *I_sc_*. The current passed through the p-n junction, causing the diffusion current loss *I_d_* [19]. The current passing through the load was:

(1) $$I_{L}=I_{\mathrm{SC}}-I_{d}$$

An ideal diode equation estimated the *I_d_* value:

(2) $$I_{d}=\frac{I_{\mathrm{SC}}}{A\cdot e^{\frac{{\mathrm{qV}}_{\mathrm{OC}}}{\mathrm{KT}}-1}}$$

In Equation (2), *q* is electronic charge, *V_oc_* is open-circuit voltage, *A* is a constant factor of voltage with a value of one in positive bias and two in negative bias, *K* is the Boltzmann constant, and *T* is absolute temperature. Current passing through the photovoltaic cell circuit caused a loss of *I_d_* and a rise in temperature.

**Figure 3.**
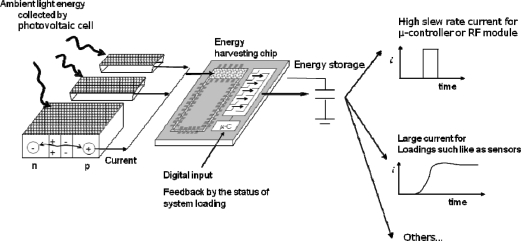
The photovoltaic cell with energy harvesting chip circuit provided a high slew rate current and steady current output.

The power generating rate of photovoltaic cells with energy harvesting chips provided an efficient power supply for the WSN nodes. A micro-electro-mechanical system (MEMS) consisting of a micro-solenoid and a ferrite core filled with nanoferrofluids had no air gap and low iron loss, increasing the power conversion efficiency to more than 95% [20]. A comparison with photovoltaic cells shows that using energy harvesting chips to harvest ambient light energy by connecting photovoltaic cell circuits in a parallel manner was 10% more efficient, and energy harvesting chip enhanced the high slew rate current, as Figure 3 shows.

Table 2 lists the measurement range and power consumptions of each wireless sensor node [21–23]. Experiment measurements indicate that the sensor nodes power output was 150 mW. The illumination of a convenience store is typically 1,000 lux, which is more than enough to provide power wireless sensor nodes. This architecture is the technical foundation of this study.

### 2.2. WSN Wireless Data Transmission

Wireless communication networks can sometimes lose communication data due to electromagnetic interference. Though this study uses wireless charging for sensor nodes, interior lighting can be shaded by customer movements, causing malfunctions in remote nodes and a loss of measurement data. Thus, a maximum entropy method was adopted at the back end of the communication module to receive sensor node data and integrate the data. The maximum entropy method is based on relevant measured values and can be decided by previous measured values or by errors in white noise patterns, as Equation (3) shows [24]:

(3) $$X_{n}=-\sum_{m-1}^{M}a_{m}X_{n-m}+E_{n}$$

In Equation (3), *E_n_* is the error value in the white noise pattern, *X_n−m_* is the previously measured value, *a_m_* is the weighting coefficient, *X_n_* is the relevant measure value, and *M* is the number of predicted data points.

Multiplying all items in Equation (3) by *X_n_*:

(4) $$X_{n}\cdot X_{n}+a_{1}X_{n-1}\cdot X_{n}+a_{2}X_{n-2}\cdot X_{n}+\cdots +a_{M}X_{n-M}\cdot X_{n}=E\cdot X_{n}$$

Equation (4) expands *n* times on time; giving an expanded polynomial as below:

(5) $$C_{\mathrm{xx}}(0)+a_{1}C_{\mathrm{xx}}(1)+a_{2}C_{\mathrm{xx}}(2)+\cdots +a_{M}C_{\mathrm{xx}}(M)=P$$

In Equation (5), *C_xx_* is the discrete autocorrelation of *X* with the time migration from 0 to *M*, and *P* is the estimated error power. Expanding *X_n−k_* migration upon time instead of all items in Formula (3) leads to the following results:

(6) $$C_{\mathrm{xx}}(k)+a_{1}C_{\mathrm{xx}}(k-1)+a_{2}C_{\mathrm{xx}}(k-2)+\cdots +a_{M}C_{\mathrm{xx}}(k-M)=0$$

A comparison of Equation (5) and Equation (6) shows that white noise caused errors in the relevant measurement, and the method of multiplying measurements also multiplied the error. Previously, the error yielding correlation in white noise patterns was zero. Combining Equation (5) and Equation (6) and expanding the formulas on time migration k yields the following matrix:

(7) $$\left[\begin{array}{cccc} C_{\mathrm{xx}}(0) & C_{\mathrm{xx}}(1) & \cdots & C_{\mathrm{xx}}(M)\\ C_{\mathrm{xx}}(1) & C_{\mathrm{xx}}(0) & \cdots & C_{\mathrm{xx}}(M-1)\\ \vdots & \vdots & & \vdots \\ C_{\mathrm{xx}}(M) & C_{\mathrm{xx}}(M-1) & \cdots & C_{\mathrm{xx}}(0) \end{array}\right]\left[\begin{array}{c} 1\\ a_{1}\\ \vdots \\ a_{M} \end{array}\right]=\left[\begin{array}{c} P\\ 0\\ \vdots \\ 0 \end{array}\right]$$

Equation (7) obtains all weighting coefficients. If any measurement parameters were lost within a certain time period, they were estimated using the following equation:

(8) $$f_{p}=\sum_{i=0}^{M}a_{i}X_{n-i}\left(M+1\leq n\leq N,\;\;a_{0}=1\right)$$

In Equation (8), *f_p_* is the forward prediction, *M* is the number of predicted data points, *a_i_* is the weighting coefficient (assuming the initial value is 1), and *X_n−i_* is the previously measured value; the boundary starts *M* + 1 until *N*.

The term *f_p_* predicts missing data when sensor nodes fail to collect temperature, relative humidity, illumination, or air flow. After recording data, the proposed model considers error estimation and adjusts continually. This system identification and autocorrelation during calculation removes noise interference. The calculation results in this study achieved precise data estimation.

### 2.3. WSN PMV Measurement

The PMV calculation method uses distributed sensor nodes to count the number of customers based on customer clothing and activity. PMV includes six parameters: temperature, irradiation temperature, relative humidity, air flow, clothing and activity, and appears as Equation (9) below [25–29]:

(9) $$\mathrm{PMV}=0.303e^{-0.36M}+0.028\left\{\begin{array}{l} \left(M-W\right)\\ -3.05\times 10^{-3}[5733-6.99(M-W)]-P_{a}\\ -0.42[(M-W)-58.15]\\ -1.7\times 10^{5}M\left(5867-P_{a}\right)\\ -0.0014M\left(34-T_{a}\right)\\ -3.96\times 10^{-8}\;f_{\mathrm{cl}}[{\left(T_{\mathrm{cl}}+273\right)}^{4}-{\left(T_{r}+273\right)}^{4}]-f_{\mathrm{cl}}h_{c}\left(T_{\mathrm{cl}}-T_{a}\right) \end{array}\right\}$$

in which:

$$\begin{array}{l} T_{\mathrm{cl}}=35.7-0.028(M-W)-I_{\mathrm{cl}}\{3.96\times 10^{-8}f_{\mathrm{cl}}[{\left(T_{\mathrm{cl}}+273\right)}^{4}-{\left(T_{r}+273\right)}^{4}]-f_{\mathrm{cl}}h_{c}\left(T_{\mathrm{cl}}-T_{a}\right)\}\\ h_{c}=\langle \begin{array}{ll} 2.38{\left(T_{\mathrm{cl}}-T_{a}\right)}^{0.25} & \;\;\mathrm{for}\;\;2.38{\left(T_{\mathrm{cl}}-T_{a}\right)}^{0.25}\;\;\geq 12.1\sqrt{V_{a}}\\ 12.1\sqrt{V_{a}} & \mathrm{for}\;\;2.38{\left(T_{\mathrm{cl}}-T_{a}\right)}^{0.25}\;\;\leq 12.1\sqrt{V_{a}} \end{array}\\ f_{\mathrm{cl}}=\langle \begin{array}{ll} 1.00+1.29I_{\mathrm{cl}} & \;\;\;\;\mathrm{for}\;\;I_{\mathrm{cl}}\geq 0.078m^{2}\;{}^{\circ}CW^{-1}\;\\ 2.1\sqrt{V_{a}} & \;\;\;\;\mathrm{for}\;\;I_{\mathrm{cl}}\leq 0.078m^{2}\;{}^{\circ}CW^{-1} \end{array} \end{array}$$

In Equation (9), *f_cl_* is the surface coefficient of clothes, *M* is the metabolic rate of human bodies (*W m^−2^*), *W* is the outward work rate (*Wm^−2^*), *I_cl_* is the insulation value of clothing (*m^2^*°C*W*^−^*^1^*), *P_a_* is partial vapor pressure (*P_a_*), *T_a_* is room temperature (°C), *T_r_* is the average temperature of irradiation (°C), *V_a_* is air flow (*m^−1^*), *h_c_* is the convective heat loss coefficient (*Wm^−2^*°C^−1^), and *T_cl_* is the clothing surface temperature (°C).

Distribution sensor nodes record data and measure temperature, relative humidity, air flow, and radiation temperature. Activities (for instance, walking) and clothing (as worn during the summer) are based on ISO 7730 [26]. The aforementioned equations revealed that the distributed PMV index ranged from +3 (hot) to −3 (cold), as Table 3 shows.

**Figure 4.**
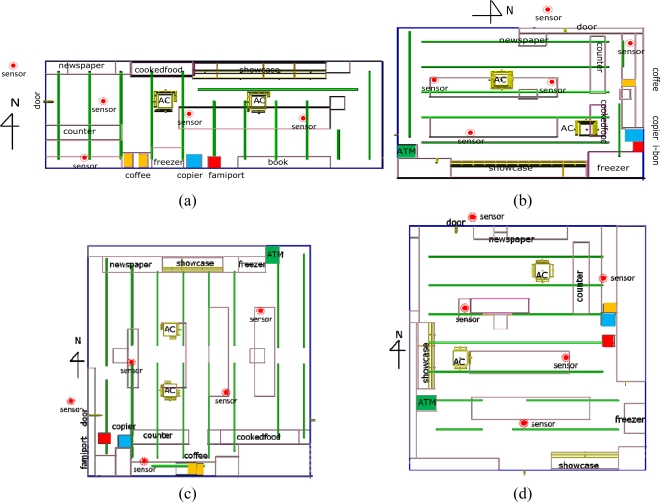
The locations where the sensor nodes were installed: (**a**) Store A; (**b**) Store B; (**c**) Store C; (**d**) Store D.

The WSNs collected physical measurements such as temperature, relative humidity, illumination, and air flow from each sensor node. Wireless sensor nodes were placed at intersections where cool air emitted from ceiling air conditioning vents and showcases mixed with hot air emitted from a cooking zone, coffee machines, and a store entrance, as Figure 4 shows. The cold and hot intersections were the locations where humans felt the largest difference. Through wireless communication, the system acquired the PMV index to achieve optimal thermal comfort in convenience stores.

**Figure 5.**
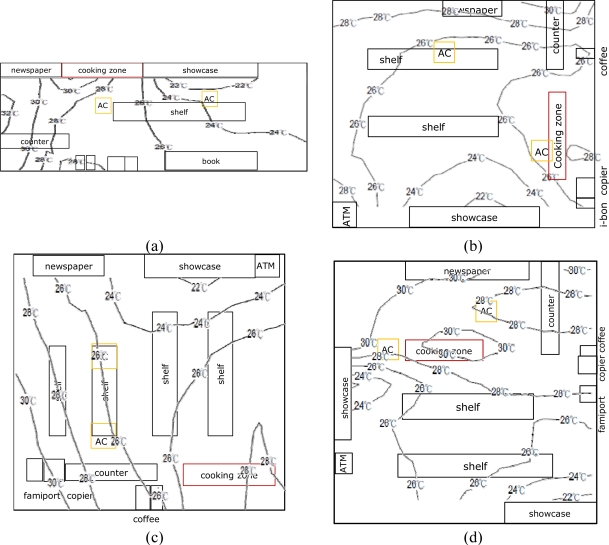
Temperature distribution schematic: **(a**) Store A; (**b**) Store B; (**c**) Store C; (**d**) Store D.

Figure 5 shows the average summer indoor temperature distribution of the four convenience stores participating in this study. The results in Figure 5 were calculated by CFD simulation, based on temperature measurements from each wireless sensor node in four convenience stores. Simulation temperature boundaries were determined according to the aforementioned collection information.

## 3. Application of CFD to Convenience Stores

The experiments in this study measured energy conservation results before and after installing the WSNs in convenience stores where both WSNs and CFD were adopted. The experiments in this study used Airpak 3.0 [30], a CFD simulation program for heating, ventilation, and air conditioning (HVAC). Table S1 shows the governing equations for this program. The convenience store indoor temperature variable affected the range of PMV indexes. In this study, heat source and cold air were established as parameters in the CFD simulation. The size of convenience stores and the heat sources are generated from outdoor air, coffee machines, a photocopier, a multi-function business machine, a refrigerator, cooking zone, and an ATM. The cold air comes from two air conditioning units.

### 3.1. Applying CFD to Convenience Stores

**Figure 6.**
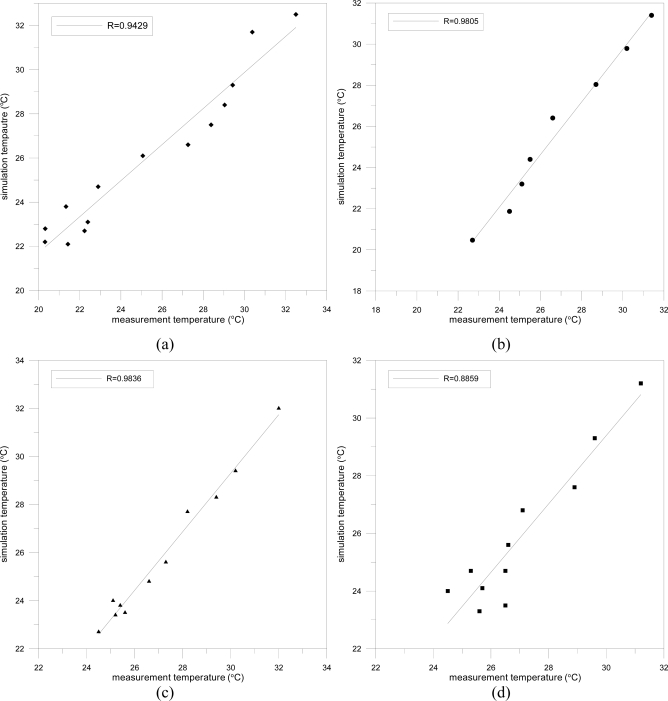
Correlation between CFD and real measurements in: (**a**) Store A; (**b**) Store B; (**c**) Store C; (**d**) Store D.

As Figure 6 shows, the experiments in this study applied CFD to four convenience stores and performed comparative analyses to judge the correlation, simulation and measurement results. The experimental setup used a linear measurement of temperature points from the entrance of stores, with one meter intervals, and a height of 1.5 meters from the ground as measurement points. The correlation *R* values of the four convenience stores ranged from 0.8859 ∼ 0.9836, demonstrating that the simulation was relatively accurate and reliable.

**Figure 7.**
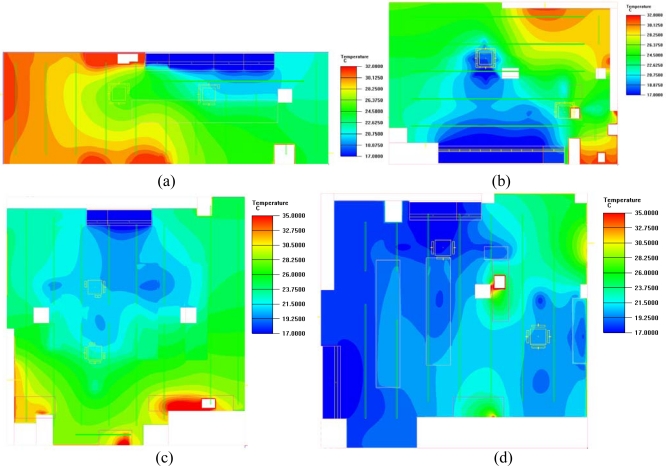
Indoor temperature distribution in convenience stores without a WSN at noon: (**a**) Store A; (**b**) Store B; (**c**) Store C; (**d**) Store D.

**Figure 8.**
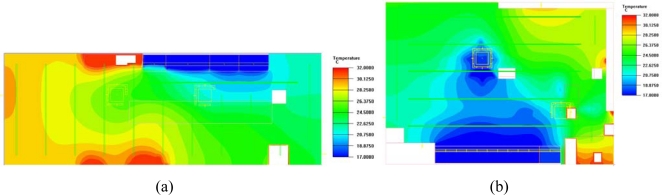

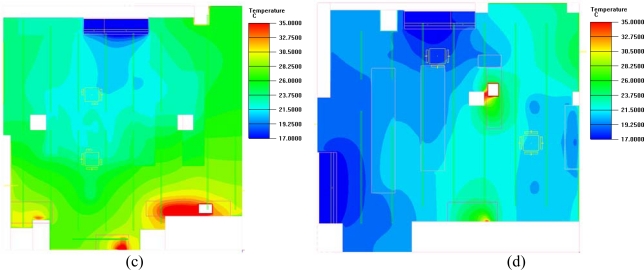
Indoor temperature distribution in convenience stores without a WSN at night: (**a**) Store A; (**b**) Store B; (**c**) Store C; (**d**) Store D.

Each convenience store was influenced differently by the store location, orientation, and the distribution of equipment in the store. These variables produced different indoor temperature distributions, as shown in Figures 7 and 8, which illustrate the simulated day and night distribution of indoor temperatures for the four stores prior to installing a WSN.

Figure 7(a) shows that the indoor temperature distribution in Store A was the highest among the four stores. Limiting the power consumption of air conditioning could save energy, but the PMV index at noon was +0.8, indicating an uncomfortable environment. Thus, power conservation was only adopted under comfortable conditions to avoid making customers feel uncomfortable. Stores B, C, and D had cooler, more comfortable conditions. Air conditioning power management was necessary to achieve a comfortable and energy saving shopping environment.

### 3.2. Applying WSN and CFD to the Energy Conservation of Air Conditioning in Convenience Stores

Figure 4 shows the distribution of WSN sensor nodes. The nodes were placed at intersections of cool and warm air, such as doors, a cooking zone, and a showcase zone, to determine the energy saving effects by day and night.

A comfortable environment in convenience stores is influenced by the number of customers, showcases, appliances in the cooking zone, and solar radiation. To maintain comfortable conditions, convenience stores should implement energy conservation practices to suit real store situations, in addition to using inverter air conditioning in compliance with governmental policy [31].

**Figure 9.**
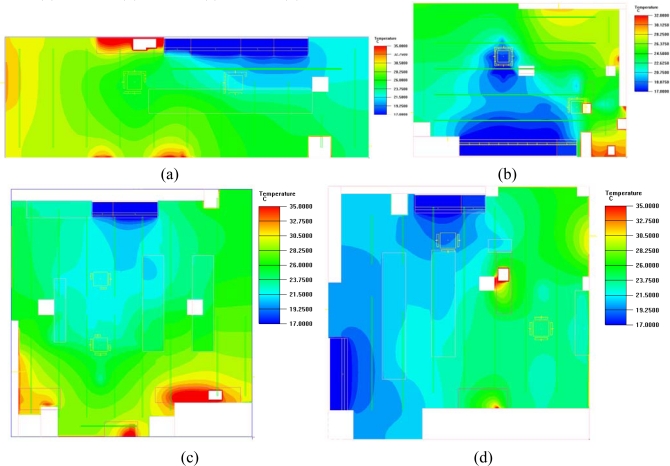
Room temperature distribution in convenience stores with WSN at noon: (**a**) Store A; (**b**) Store B; (**c**) Store C; (**d**) Store D.

**Figure 10.**
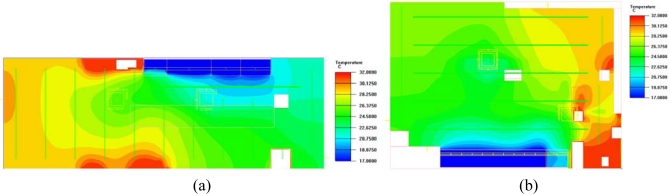

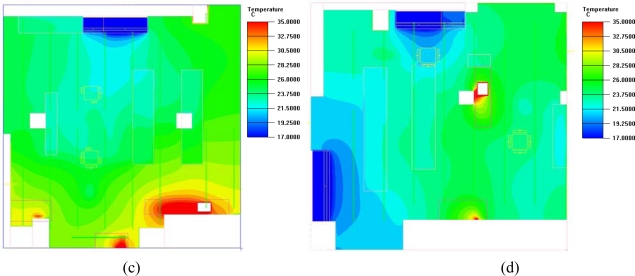
Room temperature distribution in convenience stores with WSN at night: (**a**) Store A; (**b**) Store B; (**c**) Store C; (**d**) Store D.

**Figure 11.**
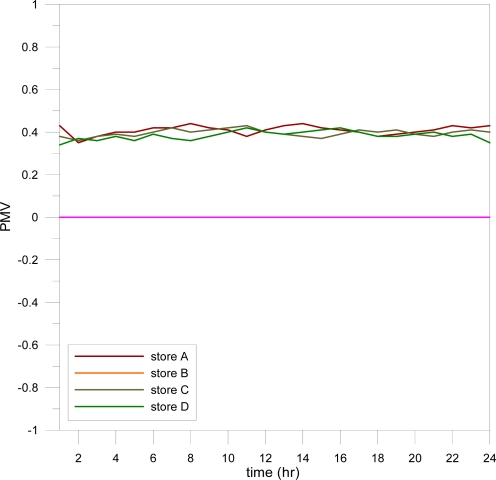
Comparison of average daily PMV in four convenience stores.

Figures 9 and 10 present the CFD results for the WSNs installed in the convenience stores at noon and night. Unlike the results in Figures 7 and 8, store A had more air flow and a lower indoor temperature at noon, while stores B, C, and D had higher indoor temperatures. Due to the presence of fewer customers at night, the WSN turned the air conditioning located near the showcase zone to fan mode only, and set the other air conditioning to a lower fan speed and 26 °C. The indoor temperature was controlled at approximately 24 to 26 °C. Figure 11 demonstrates that the PMV values of four convenience stores with WSN maintained a neutral value +0.4, which is a comfortable and energy-conserving environment.

**Figure 12.**
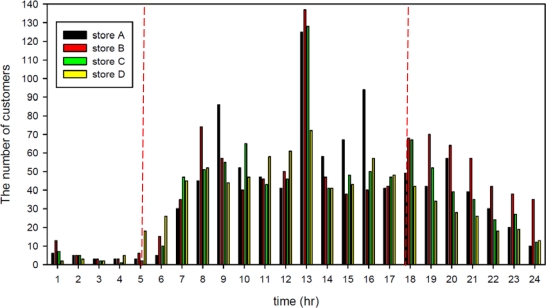
The number of customers in the four convenience stores.

**Figure 13.**
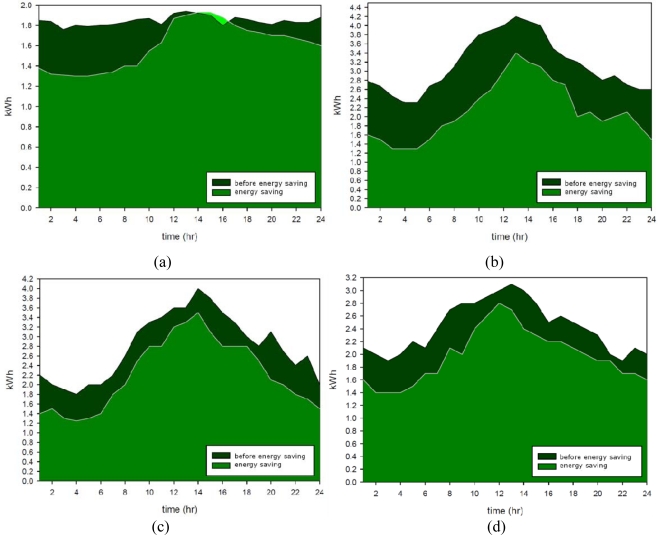
Increase in power conservation after applying a WSN in: (**a**) Store A; (**b**) Store B; (**c**) Store C; (**d**) Store D.

Figure 12 shows that the customers in the four convenience stores were primarily distributed from 8:00 am to 8:00 pm, with crowds peaking at noon and decreasing at night. The electronic power usage of air conditioning equipment in four convenience stores was limited by the WSN. Figure 13 shows the daily average of summer power conversation efficiency for the WSNs installed in the stores, indicating that the number of customers affected the air conditioning power consumption. The experimental data further showed that power usage peaks at noon, implying that the power usage of air conditioning is affected by the sunset and the daily weather. Compared 2008 data with 2007 data, the power usage of Stores A, B, C and D decreased by 10%, 32%, 21%, and 19 % respectively after installing the WSN.

## 4. Analysis of Marginal Energy Conservation Benefit of WSN

**Figure 14.**
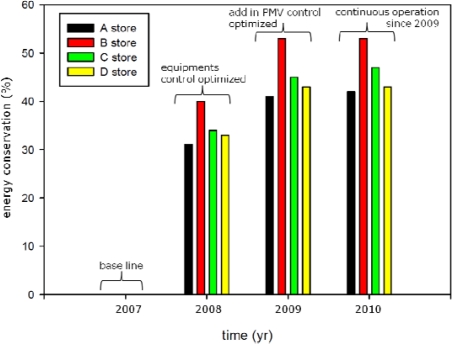
The efficiency of energy conservation of air conditioning in a convenience store. 2007 was the baseline (with no WSN), with 2008 to 2010 figures indicating the percentage of energy conservation.

A wireless sensor network (WSN) was installed in four convenience stores in Taipei City in 2008 to record indoor temperature, relative humidity, air velocity, and illumination values. Figure 14 shows that for Store B, air conditioning optimization achieved a 40% increase in energy conservation compared with the 2007 baseline. Additions to the set-up continued beyond 2008, adding the thermal comfort and crowds peak, off-peak features to achieve human-friendly energy savings in 2009.

Under this condition, the system achieved 53% air conditioning energy conservation. In 2010, the same operating method as 2009, the energy conservation was again 53%. These experiment results show that 53% was the maximum energy conservation for store B, and the highest marginal energy conservation benefit among all four stores.

**Figure 15.**
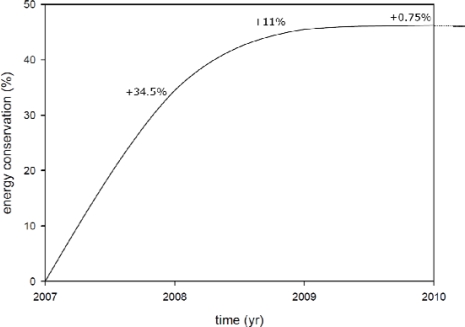
The schematic of the marginal energy conservation benefit of four convenience stores with a WSN.

Figure 15 shows that the air conditioning energy conservation for all four convenience stores in 2008 had increased 34.5% from 2007 and 11% from 2008 to 2009; however, the air conditioning energy conservation in 2010 only increased 0.75% and its trend curve approached a horizontal line. This reveals a limit to the growth of air conditioning power conservation.

Table 4 shows the cost of installing a wireless sensor network in a convenience store. Each convenience store requires three temperature sensors, two humidity sensors, two air velocity sensors, one illumination sensor, and one gateway. The basic cost is 500 US dollars, and depending on how the sensor nodes are arranged, the cost-recovery period is 3 to 6 months.

**Figure 16.**
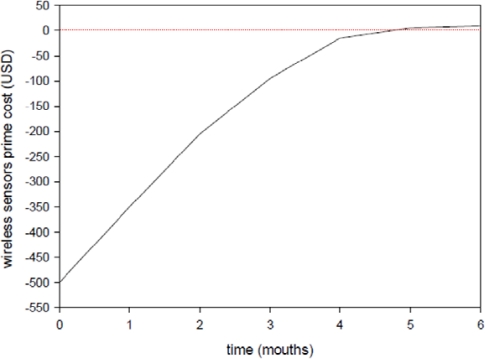
The cost-recovery period after installing a WSN.

Figure 16 shows that the proposed wireless sensor networks have been functioning since July 2008. The first four months, July to October, exhibited the greatest cost-recovery, amounting to almost full cost-recovery. Though the cooler weather in the fifth month caused a cost-recovery slowdown, most of the costs had already been recovered.

## 5. Conclusions

The experiments in this study prove that WSNs monitoring air conditioning in convenience stores have successfully achieved energy conservation since 2008. The proposed multiple-node wireless sensor networks (WSNs) reduced the electric power usage of air conditioning by 20% in summer, based on optimal operation and crowd-peak or off-peak conditions. The thermal comfort of the predicted mean vote (PMV) was +0.4, reflecting a comfortable yet energy-conserving shopping environment.

This study uses the Airpak CFD software to calculate and simulate the indoor temperature distribution, and used these results to analyze the placement of equipment where in the convenience store in relation to the store entrance. This study also determines that the marginal energy conservation benefits for the four convenience stores that installed a WSN in 2008 was that the average air conditioning equipment power conservation was 45.5% per year. The cost of the WSN installations was approximately 500 US dollars. The cost-recovery period ranged from 3 to 6 mouths, depending on the size of the convenience store. This results show that the proposed WSN technique is a great strategy for achieving energy conservation and a comfortable shopping environment.

## Figures and Tables

**Table 1.** Comparison of stray energy in the air.

**Type of stray energy**	**Source**	**Recovery mechanism**	**Estimation the produced energy density (cm^2^)**
Thermal sources	Air conditioning cooling decreases temperature (summer), or heat pump generates heat (winter). Due to above condition, this caused temperature difference between indoors and outdoors	Thermoelectric chip	15 μW/10 °C
Vibration sources	Vibration due to human activities (walking, running, *etc.*) and machine tools	Piezoelectric Electrostatic Electromagnetic	200 μW 50–100 μW 1 μW
Light sources	Solar radiation and indoor lighting	Photovoltaic cell	**sunlight** direct sunlight: 15 mw oblique sunlight: 150 μw **indoor lighting (500–1,500 lux)** direct light: 5–15 mw oblique light: 50–150 μw

**Table 2.** The power consumption of each sensor and wireless sensor node (powered by energy harvesting).

**Item**	**Type**	**Measurement range**	**Power supply**	
Temperature sensor	LM355	−55 °C ∼ +150 °C	300 μA at 3 V	
Relative humidity sensor	SHT1X	20% ∼ 80%	550 μA at 3 V	
Photosensor with signal processing circuits		Accuracy level was ±2% of the reading range, which yielded 1.5 to 4.5 °C uncertainty.	1 mA at 2.5 V	
MEMS flow sensor developed by this lab		0.1 ms^−1^ ∼ 0.45 ms^−1^	5 mA at 2.5 V	
Signal processor and RF transmission module	PIC16F526		0.5 ∼ 10 mA at 3.5 V	Nominal power: 20 mW Standby power: 1.75 mW Transient power max: 35 mW
Photovoltaic cell	SC 7035			

**Table 3.** Different thermal comfort levels represented by different PMV index levels.

**Index**	**Body sensation**
+3	hot
+2	warm
+1	slightly warm
0	neutral
−1	slightly cool
−2	cool
−3	cold

**Table 4.** Sensor component cost.

**Item**	**Type**	**Cost (USD)**	**Cost of a set of sensors (USD)**
Temperature sensor	LM355	3	18
Relative humidity sensor	SHT1X	6.5	21.5
Photosensor with signal processing circuits		0.5	15.5
MEMS flow sensor developed by this lab		30	45

Total cost of installing the WSN (including 3 temperature sensors, 2 relative humidity sensors, 2 air flow sensors, 1 illumination sensor, and 1 gateway)			500

**Table S2.**
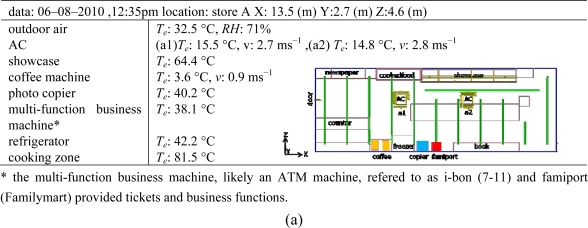

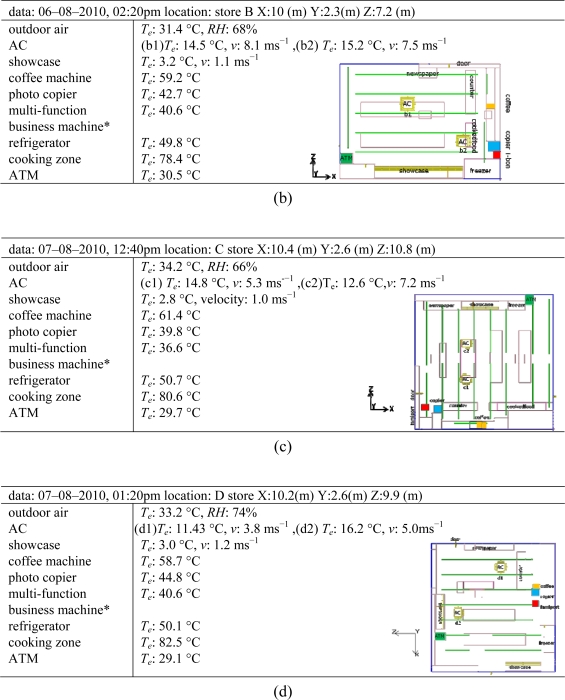
Values of ventilation and machines heat dissipation in convenience stores.

## Nomenclature:

**Photovoltaic cell**
*I_sc_*: Closed-circuit voltage (*V*)
*I_d_*: Diffusion current loss (*I*)
*V_oc_*: Open-circuit voltage (*V*)
*A*: Constant factor of voltage
*K*: Boltzmann constant
*T*: Absolute temperature (*K*)

**Predicted mean vote (PMV)**
*f_cl_*: Surface coefficient of clothes
*M*: Metabolic rate of human bodies (*Wm^−2^*)
*W*: Outward work rate (*Wm^−2^*)
*I_cl_*: Insulation value of clothing (*m^2^°CW^−^^1^*)
*P_a_*: Partial vapor pressure (*Pa*)
*T_a_*: Room temperature (°*C*)
*T_r_*: Average temperature of irradiation (°*C*)
*V_a_*: Air flow (*m^−1^*)
*h_c_*: Convective heat loss coefficient (*Wm^−2^*°*C^−1^*)
*T_cl_*: Clothing surface temperature (°*C*)

**Measurement value**
*T_e_*: Measurement temperature (°*C*)
*v*: Measurement air flow (*ms^−1^*)
*RH*: Measurement relative humidity (*%*)

## Appendix

**Table S1.** Governing equations with two-equation turbulence model.

Equations	*φ*	Γ*_φ_*	*S_φ_*
Continuity	1	0	0
Momentum Energy	*V⇀*	*μ_eff_*	− ∇*_p_* − *ρ*_0_*β*(*T* − *T*_0_)*g⇀*
	*h*	*λ_eff_ C_p_*^−1^	0
Turbulence kinetic energy	*κ*	*μ_eff_ σ_κ_*^−1^	*P_k_* + *G_b_* − *ρɛ*
Turbulence kinetic energy	*ɛ*	*μ_eff_ σ_ɛ_*^−1^	(*C*_1_*ɛP_k_* − *C*_2_*ɛ*^2^ + *C*_3_*ɛG_b_*)*k*^−1^

Dissipation rate	

Equations	constants
*h* = *C_p_* (*T* − *T_ref_*)	*C*_1_ = 1.44
*P_k_* = *μ_t_* (*U_ij_* + *U_ji_*)*U_ij_*	*C*_2_ = 1.92
*G_b_* = *βg_i_μ_t_∂T*(Pr*_t_∂x_i_*)^−1^	*C*_3_ = *C*_1_ tanh|*νu*^−1^|
*μ_eff_* = *μ* + *μ_t_*	*C_μ_* = 0.09
	*σ_κ_* = 1.0
*λ_eff_* = *λ* + *λ_t_*	*σ_ɛ_* = 1.3
*D_eff_* = *D* + *D_t_*	Pr*_t_* = 0.85
*D_t_* = *μ_t_* (*ρSc_t_*)^−1^	*Sc_t_* = 0.7
